# Transcriptome analysis reveals genes associated with late blight resistance in potato

**DOI:** 10.1038/s41598-024-60608-3

**Published:** 2024-07-05

**Authors:** Nisha Bhatia, Jagesh Kumar Tiwari, Chandresh Kumari, Rasna Zinta, Sanjeev Sharma, Tanuja Buckseth, Ajay K. Thakur, Rajesh K. Singh, Vinod Kumar

**Affiliations:** 1 ICAR-Central Potato Research Institute, Shimla, Himachal Pradesh India; 2 School of Biotechnology, Shoolini University, Solan, Himachal Pradesh India; 3 ICAR-Indian Institute of Vegetable Research, Varanasi, Uttar Pradesh India; 4 School of Bioengineering and Biosciences, Lovely Professional University, Phagwara, Punjab India

**Keywords:** Biotechnology, Molecular biology, Plant sciences

## Abstract

Late blight is a serious disease of potato worldwide. Our study aimed to unveil genes involved in late blight resistance in potato by RNA-seq analysis after artificial inoculation under controlled conditions. In this study, two potato somatic hybrids (P7 and Crd6) and three varieties such as Kufri Girdhari, Kufri Jyoti and Kufri Bahar (control) were used. Transcriptiome analysis revealed statistically significant (*p* < 0.05) differentially expressed genes (DEGs), which were analysed into up-regulated and down-regulated genes. Further, DEGs were functionally characterized by the Gene Ontology annotations and the Kyoto Encyclopedia of Genes and Genomes pathways. Overall, some of the up-regulated genes in resistant genotypes were disease resistance proteins such as CC-NBS-LRR resistance protein, ankyrin repeat family protein, cytochrome P450, leucine-rich repeat family protein/protein kinase family, and MYB transcription factor. Sequence diversity analysis based on 38 peptide sequences representing 18 genes showed distinct variation and the presence of three motifs in 15 amino acid sequences. Selected genes were also validated by real-time quantitative polymerase chain reaction analysis. Interestingly, gene expression markers were developed for late blight resistant genotypes. Our study elucidates genes involved in imparting late blight resistance in potato, which will be beneficial for its management strategies in the future.

## Introduction

Late blight, caused by the oomycetes *Phytophthora infestans*, is the most serious disease of potato. This pathogen is a highly variable and severely damages potato crops, and therefore its management is a challenging task. During the 1960s–1980s, race-specific resistance genes were deployed in potato breeding using the hexaploid wild species *Solanum demissum*^1^. However, over the decades, the *R* genes were defeated due to the emergence of new *P. infestans* strains. Hence, there is a need to identify new resistance sources in wild species background, of which many of them are yet to be characterized at transcriptome level.

The genus *Solanum* is a rich source of genetic diversity, containing over 200 wild species^2^. Several wild species have been identified in potato, which confer late blight resistance such as *S. pinnatisectum*, *S. cardiophyllum*, *S. bulbocastanum*, *S. stoloniferum, S. bulbocastanum*, *S. demissum*, *S. polytrichon*, and *S. microdontum*^3^. Many useful genes derived from wild species cannot be transferred through conventional breeding due to sexual barriers caused by differences in the ploidy number and the endosperm balance number^4^. Hence, somatic hybridization has been deployed via protoplast fusion to overcome crossing barriers^5^. For example, we developed interspecific potato somatic hybrids through protiplast fusion for late blight resistance namely P7 (*Solanum tuberosum* + *S. pinnatisectum*)^6^, and Crd6 (*S. tuberosum* + *S. cardiophyllum*)^7^. However, these somatic hybrids have not yet characterized at transcriptome level for late blight resistance genes.

With the advancement in the post-genomics era and availability of the potato genome^8^, it is now feasible to analyse genes at the whole genome level in potato. Many reports are available on whole transcriptome sequencing for late blight resistance in potato^9–11^. Transcriptomics studies have been performed on host–pathogen interaction to discover gene networks regulating late blight resistance in potato^12^. In addition, many candidate genes have been identified in potato for multiple traits such as late blight, potato virus Y and bacterial wilt causing pathogens interaction^13^, wild species *S. pinnatisectum* conferring late blight resistance^14^, contrasting potato foliage and tuber defense response^15^, and scion grafted to potato rootstock for improving late blight resistance^16^.

In this study, we aimed to identify genes associated with late blight resistance by transcriptome sequencing in interspecific potato somatic hybrids and varieties after artificial inoculation. Differentially expressed genes (DEGs), heat map, Venn diagram, scatter plot, volcano plots, Gene Ontology (GO) characterization, the Kyoto Encyclopedia of Genes and Genomes (KEGG) pathways analysis were investigated. Selected genes likely to be involved in the resistance mechanism were characterized at the sequence level and motifs were discovered. A few selected DEGs were confirmed by real-time quantitative polymerase chain reaction (RT-qPCR) analysis. Our study sheds light on genes involved in late blight resistance in potato that can be used for its management through breeding and biotechnological interventions.

## Results

### Late blight resistance assay

**Figure 1:**
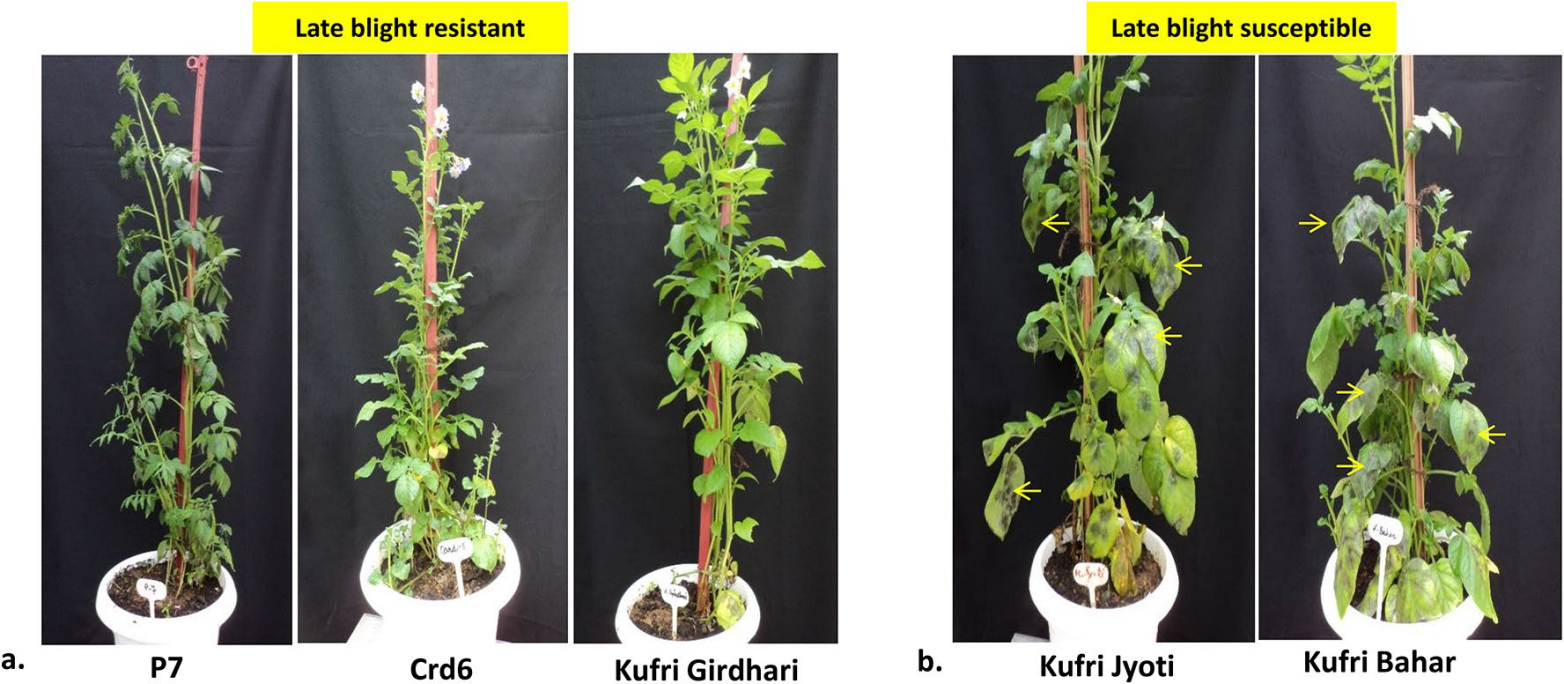
Late blight resistance assay of potato somatic hybrids and varieties under contorlled conditions by artificial inoculation with *P. infestans*: (**a**) highly resistant (P7, Crd6, and Kufri Girdhari), (**b**) susceptible (Kufri Jyoti and Kufri Bahar). Late blight infected leaves (black lesions) in susceptoble genotypes (Kufri Jyoti and Kufri Bahar) are shown with yellow arrow.

Five potato genotypes were tested twice for late blight resistance by artificial inoculation of *P. infestans* under controlled conditions (Fig. 1). Based on the mean data of two years, we identified highly resistant genotypes such as somatic hybrids P7 (Area Under Disease Progressive Curve, AUPDC: 32.89) and Crd6 (AUPDC: 2.75), and potato variety cv. Kufri Girdhari (AUPDC: 1.12). Whereas, a popular potato cv. Kufri Jyoti (AUPDC: 167.54) was susceptible compared to highly susceptible cv. Kufri Bahar (control) (AUPDC: 241.33) (Table 1). Resistant and susceptible genotypes are shown in Fig. 1.

**Table 1:** Late blight resistant test of potato somatic hybrids and varieties by artificial inoculation of *P. infestans* under controlled conditions.

Genotype	Late blight infection (AUDPC value)			Class*
	2021	2022	Mean	
P7	37.45	28.34	32.89	HR
Crd6	0	5.50	2.75	HR
Kufri Girdhari	0	2.25	1.12	HR
Kufri Jyoti	156.34	178.75	167.54	S
Kufri Bahar	234.67	248.0	241.33	S
CD (*p* < 0.05)	6.98	9.30	8.24	

*Class was determined based on the area under disease progressive curve (AUDPC) values: highly resistant (HR ≤ 50), resistant (R = 50–100), moderately resistant (MR ≥ 100–150), susceptible (S ≥ 150).

### Transcriptome data generation

Total RNA-sequencing of five samples was performed in duplicates based on 2 × 150 bp chemistry of Illumina NextSeq500 platform. High quality paired-end reads (QV > 25) were generated for all genotypes in both replicates (R1/R2) such as P7 (10./8.72 Gb), Crd6 (8.88/8.21 Gb), Kufri Girdhari (11.13/9.51 Gb), Kufri Jyoti (9.92/10.23 Gb), and Kufri Bahar (10.77/9.62 Gb). The reference mapping of the quality reads showed good mapping results with the potato genome sequence data for all samples in bot replicates (R1/R2) namely P7 (75.3%/78.3%), Crd6 (72.7%/70.2%), Kufri Girdhari (85.8%/81.56%), Kufri Jyoti (82.9%/85.32%) and Kufri Bahar (75.3%/81.21%).

### Identification of differeltially expressed genes (DEGs)

**Figure 2:**
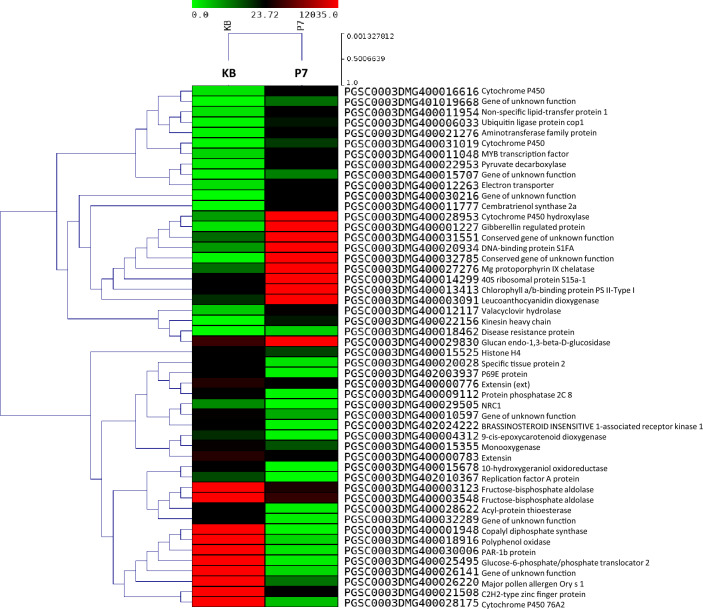
Heat maps of top 50 differentially expressed genes (*p* < 0.05) for late blight resistance in potato somatic hybrid P7 versus Kufri Bahar (KB, control) by RNA-seq. In heat map, each horizontal line refers to a gene. Relatively up-regulated genes are shown in red colour, whereas down-regulated genes are shown in green colour.

**Figure 3:**
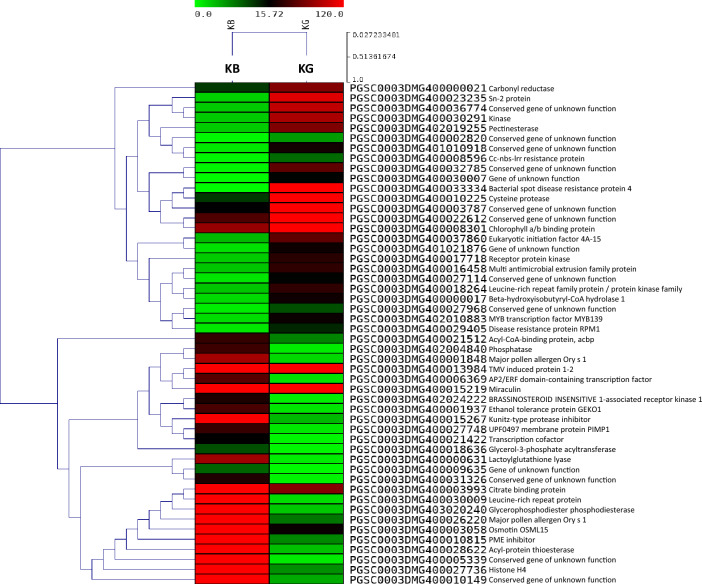
Heat maps of top 50 differentially expressed genes (*p* < 0.05) in potato variety Kufri Girdhari (KG) versus Kufri Bahar (KB, control) by RNA-seq after artificial inoculation of *P. infestans* under controlled conditions. In heat map, each horizontal line refers to a gene. Relatively up-regulated genes are shown in red colour, whereas down-regulated genes are shown in green colour.

DEGs were identified in P7, Crd6, Kufri Girdhari, and Kufri Jyoti versus Kufri Bahar (control). The complete lists of significant DEGs are provided in Supplementary Excel datasets # 1–4, and summarized in Table 2. Significant DEGs were identified based on the statistical significance (*p* ≤ 0.05) for up-regulated genes (≥ 2 log_2_ fold change (FC)) and down-regulated genes (≤ − 2 log_2_ FC). Table 3 provides a summary of top 10 DEGs of each up-regulated and down-regulated in the genotypes. Heat maps of DEGs in selected samples are shown in Fig. 2 (P7), and Fig. 3 (Kufri Girdhari, KG). Some of the highly up-regulated genes (> 3 Log_2_ FC) were over-represented in most samples were disease resistance protein, transcription factor (MYB, AP2-EREBP), leucine-rich repeat receptor kinase, CC-NBS-LRR/NBS-LRR resistance protein, fructose-bisphosphate aldolase, Sn-2 protein, zinc finger protein, late blight resistance protein homolog R1B-23 so on. Similarly, the predominant down-regulated genes (< − 3 Log_2_ FC) were C2H2-type zinc finger protein, BRASSINOSTEROID INSENSITIVE 1-associated receptor kinase 1, zinc finger protein, ethylene-responsive transcription factor 4, and protein kinase domain containing protein.

**Table 2:** Summary of differentially expressed genes (DEGs) by RNA-sequencing in potato genotypes after artificial inoculation with *P. infestans* for late blight resistance.

Genotype*	Total DEGs	DEGs (*p* < 0.05)	
		Up-regulated	Down-regulated
P7	18,529	273	300
Crd6	19,957	207	269
Kufri Jyoti	18,503	407	208
Kufri Girdhari	19,071	221	378

*Potato cv. Kufri Bahar was used as a control in the DEGs analysis.

**Table 3:** Selected differentially expressed genes (*p* < 0.05) in the leaf tissues of potato genotypes after artificial inoculation with *P. infestans* by RNA-seq analysis.

Sr. no	Gene ID	Gene description	Gene expression (Log_2_ FC)*
(i) P7			
Up-regulated			
1	PGSC0003DMG400011777	Cembratrienol synthase 2a	9.227
2	PGSC0003DMG400028953	Cytochrome P450 hydroxylase	8.903
3	PGSC0003DMG400001227	Gibberellin regulated protein	6.458
4	PGSC0003DMG400022156	Kinesin heavy chain	5.703
5	PGSC0003DMG400018462	Disease resistance protein	5.625
6	PGSC0003DMG400020934	DNA-binding protein S1FA	5.264
7	PGSC0003DMG400016616	Cytochrome P450	5.088
8	PGSC0003DMG400011048	MYB transcription factor	4.291
9	PGSC0003DMG400003123	Fructose-bisphosphate aldolase	4.046
10	PGSC0003DMG400008146	Leucine-rich repeat receptor kinase	3.613
Down-regulated			
1	PGSC0003DMG400012117	Valacyclovir hydrolase	− 7.910
2	PGSC0003DMG400015525	Histone H_4_	− 5.750
3	PGSC0003DMG400021508	C_2_H_2_-type zinc finger protein	− 7.712
4	PGSC0003DMG400001948	Copalyl diphosphate synthase	− 8.294
5	PGSC0003DMG400025495	Glucose-6-phosphate/phosphate translocator 2	− 5.355
6	PGSC0003DMG402024222	BRASSINOSTEROID INSENSITIVE 1-associated receptor kinase 1	− 4.777
7	PGSC0003DMG400030009	Leucine-rich repeat protein	− 3.703
8	PGSC0003DMG400034322	Zinc finger protein	− 3.583
9	PGSC0003DMG400005113	PR1 protein	− 3.411
10	PGSC0003DMG400006369	AP2/ERF domain-containing transcription factor	− 3.095
(ii) Crd6			
Up-regulated			
1	PGSC0003DMG400024281	Gamma aminobutyrate transaminase isoform2	6.698
2	PGSC0003DMG400011751	2-Oxoglutarate-dependent dioxygenase	6.549
3	PGSC0003DMG400020870	RNase H family protein	6.296
4	PGSC0003DMG400021796	GTP-binding protein	6.057
5	PGSC0003DMG400005931	Sterol delta-7 reductase DWF5	5.909
6	PGSC0003DMG400022263	Fructose-bisphosphate aldolase	5.161
7	PGSC0003DMG400003548	Fructose-bisphosphate aldolase	4.610
8	PGSC0003DMG400010894	Transcription factor	4.334
9	PGSC0003DMG400000340	MYB transcription factor	4.311
10	PGSC0003DMG400012167	Multidrug resistance protein ABC transporter family	2.777
Down-regulated			
1	PGSC0003DMG400003056	Ethylene-responsive proteinase inhibitor 1	− 7.287
2	PGSC0003DMG400029830	Glucan endo-1,3-beta-d-glucosidase	− 6.986
3	PGSC0003DMG400021422	Transcription cofactor	− 4.712
4	PGSC0003DMG400006369	AP2/ERF domain-containing transcription factor	− 4.673
5	PGSC0003DMG400008761	R2R3 transcription factor MYB108 1	− 4.629
6	PGSC0003DMG402029631	Pleiotropic drug resistance protein 1	− 4.417
7	PGSC0003DMG400017231	Transcription factor TSRF1	− 4.113
8	PGSC0003DMG400026821	Ethylene-responsive transcription factor 4	− 3.328
9	PGSC0003DMG400005590	Disease resistance protein	− 3.250
10	PGSC0003DMG400000211	WRKY transcription factor	− 2.656
(iii) Kufri Girdhari			
Up-regulated			
1	PGSC0003DMG400033334	Bacterial spot disease resistance protein 4	6.998
2	PGSC0003DMG400023235	Sn-2 protein	5.287
3	PGSC0003DMG400008596	Cc-nbs-lrr resistance protein	4.013
4	PGSC0003DMG400018264	Leucine-rich repeat family protein/protein kinase family protein	3.322
5	PGSC0003DMG402010883	MYB transcription factor MYB139	3.282
6	PGSC0003DMG400029405	Disease resistance protein RPM1	3.272
7	PGSC0003DMG400002426	Resistance gene	3.253
8	PGSC0003DMG400018464	Disease resistance protein	3.192
9	PGSC0003DMG401010943	Nbs-lrr resistance protein	2.451
10	PGSC0003DMG400025545	Late blight resistance protein homolog R1B-23	2.082
Down-regulated			
1	PGSC0003DMG400030009	Leucine-rich repeat protein	− 6.111
2	PGSC0003DMG403020240	Glycerophosphodiester phosphodiesterase	− 5.722
3	PGSC0003DMG402004840	Phosphatase	− 5.277
4	PGSC0003DMG400021512	Acyl-CoA-binding protein, acbp	− 5.130
5	PGSC0003DMG400006369	AP2/ERF domain-containing transcription factor	− 5.014
6	PGSC0003DMG400021422	Transcription cofactor	− 4.724
7	PGSC0003DMG401008167	AT-HSFB3 (*Arabidopsis thaliana* heat shock transcription factor B3)	− 3.554
8	PGSC0003DMG400008761	R2R3 transcription factor MYB108 1	− 3.551
9	PGSC0003DMG400017458	Transcription regulator	− 3.191
10	PGSC0003DMG400017231	Transcription factor TSRF1	− 2.544
(iv) Kufri Jyoti			
Up-regulated			
1	PGSC0003DMG400007665	Cinnamoyl-CoA reductase	6.960
2	PGSC0003DMG400016172	Leucine zipper-ef-hand containing transmembrane protein	5.605
3	PGSC0003DMG400018464	Disease resistance protein	5.122
4	PGSC0003DMG400018429	Bacterial spot disease resistance protein 4	3.545
5	PGSC0003DMG400028081	Cc-nbs-lrr resistance protein	3.405
6	PGSC0003DMG400002272	Transcription factor AP2-EREBP	2.917
7	PGSC0003DMG400000495	C2H2L domain class transcription factor	2.863
8	PGSC0003DMG400045092	Ethylene-responsive transcription factor 1B	2.744
9	PGSC0003DMG400010309	Ethylene-responsive transcription factor	2.534
10	PGSC0003DMG400008394	Cc-nbs-lrr resistance protein	2.519
Down-regulated			
1	PGSC0003DMG400020660	Protein kinase domain containing protein	− 4.898
2	PGSC0003DMG400016776	Cytochrome P450	− 4.573
3	PGSC0003DMG400030771	Alcohol dehydrogenase 3	− 4.060
4	PGSC0003DMG401015527	Serine-threonine protein kinase, plant-type	− 4.051
5	PGSC0003DMG400008677	NAC domain protein	− 3.820
6	PGSC0003DMG400033354	Sn-1 protein	− 3.383
7	PGSC0003DMG400029569	Proline-rich protein	− 2.987
8	PGSC0003DMG400010224	Phytophthora-inhibited protease 1	− 2.886
9	PGSC0003DMG400005590	Disease resistance protein	− 2.760
10	PGSC0003DMG400002427	Bacterial spot disease resistance protein 4	− 2.740

*Gene expression analysis in P7, Crd6, Kufri Girdhari and Kufri Jyoti was performed in comparison with susceptible control Kufri Bahar and expressed in term of Log_2_ fold change.

### Venn diagram analysis and gene expression marker development

**Figure 4:**
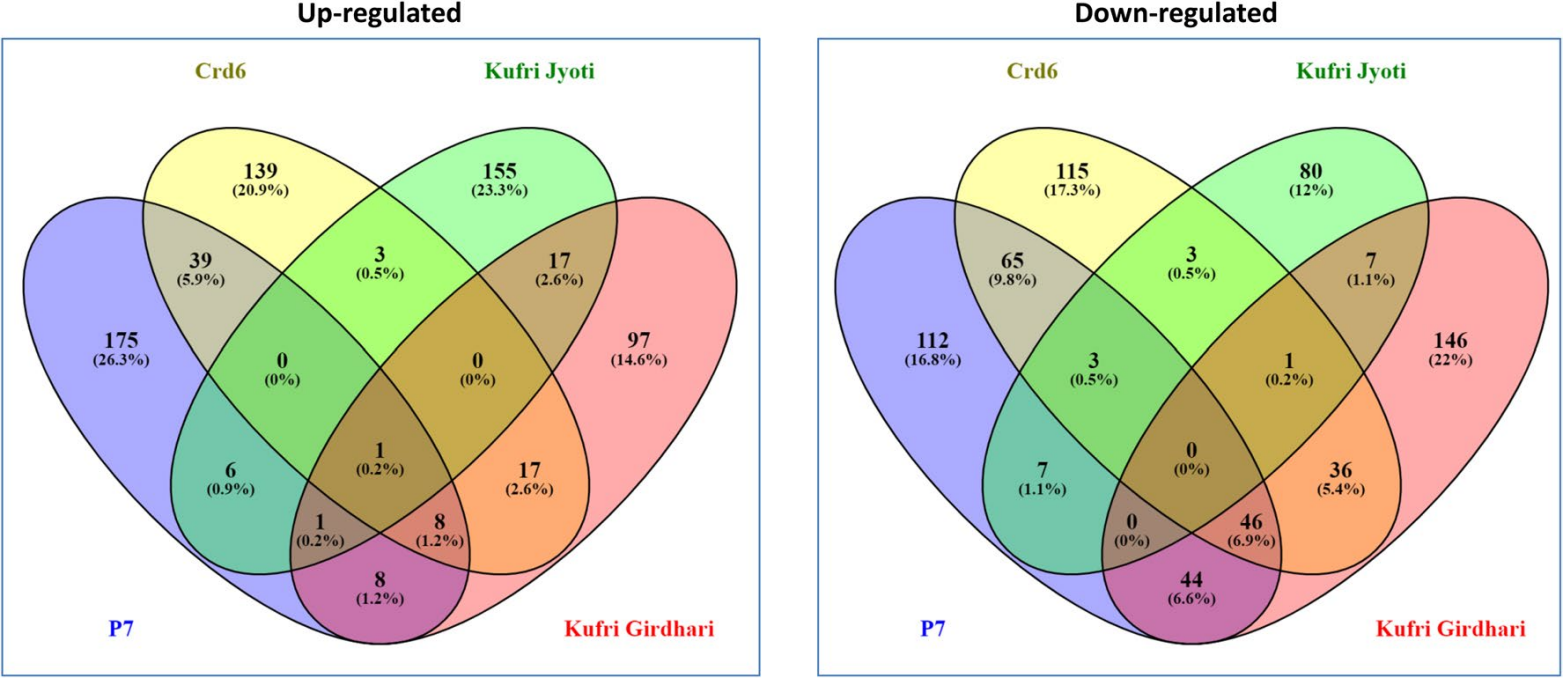
Venn diagrams showing common genes (up-regulated and down-regulated DEGs) in potato genotypes P7, Crd6, Kufri Jyoti and Kufri Girdhari.

Venn diagram analysis showed common genes in four genotypes such as P7, Crd6, Kufri Girdhari and Kufri Jyoti (Fig. 4). In which, P7 and Crd6 shared only 39 common up-regulated genes and 65 down-regulated genes. On the other hand, Kufri Girdhari and Kufri Jyoti shared 17 up-regulated and 7 down-regulated genes. A total of eight genes were found common among the resistant genotypes (P7, Crd6, and Kufri Girdhari). These genes were fructose-bisphosphate aldolase (PGSC0003DMG400022263), flavonoid glucoyltransferase UGT73E2 (PGSC0003DMG400017119), carbonyl reductase (PGSC0003DMG400000021), gamma aminobutyrate transaminase isoform2 (PGSC0003DMG400024281), chloroplast ferredoxin I (PGSC0003DMG400011950), conserved gene of unknown function (PGSC0003DMG400005633), ferric-chelate reductase (PGSC0003DMG401018223), and glyceraldehyde-3-phosphate dehydrogenase B subunit (PGSC0003DMG400029406). Of which gene expression markers (RT-qPCR) were developed based on higher gene expression in the resistant genotypes for four genes namely fructose-bisphosphate aldolase (PGSC0003DMG400022263), gamma aminobutyrate transaminase isoform2 (PGSC0003DMG400024281), carbonyl reductase (PGSC0003DMG400000021) and glyceraldehyde-3-phosphate dehydrogenase B subunit (PGSC0003DMG400029406) (Supplementary Table S1). RT-qPCR gene expression patterns were mathched with our RNA-seq results. Thus, these genes could be used in future for the identification of resistant genotypes based on RT-qPCR analysis.

### GO annotation and KEGG pathways analysis

**Figure 5:**
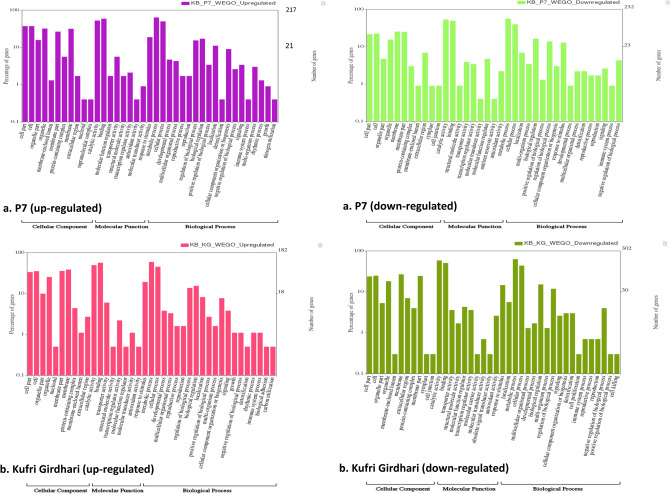
Gene Ontology (GO) characterization for cellular component, molceular fucntion, and biological process of up-regulated and down-regulated DEGs in P7 and Kufri Girdhari.

**Figure 6:**
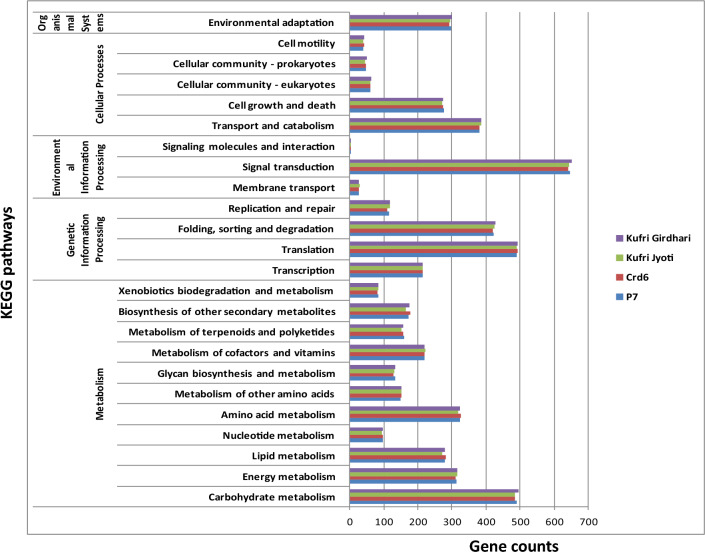
KEGG pathways classification of the annotated genes counts in potato genotypes (P7, Crd6, Kufri Jyoti and Kufri Girdhari). X-axis indicates gene counts and y-axis indicates KEGG pathways groups.

All DEGs were functionally characterized by GO terms where molecular function showed the highest gene counts (65,645) followed by biological process (54,476) and cellular component (48,301) (Supplementary Table S2). The WEGO plots are depicted in Fig. 5 for up-regulated and down-regulated genes in P7 and Kufri Girdhari (Supplementary Excel datasets # 5–8). The GO annotations of genes in CRd6 and Kufri Jyoti are shown in Supplementary Fig. S1. Overall, a few GO terms were found predominantly such as catalytic activity, binding, metabolic process, cellular process, cell, and membrane. All DEGs were classified into 24 KEGG functional pathways categories, which included KEGG annotated gene counts such as P7 (5448), Crd6 (5428), Kufri Jyoti (5414) and Kufri Girdhari (5478) (Fig. 6) (Supplementary Tables S3 and S4) and detailed in Supplementary Excel datasets (#9–12). In all combinations, maximum KEGG annotated gene counts were found for signal transduction than other pathways like translation, carbohydrate metabolism, folding sorting and degradation, amino acid metabolism, energy metabolism, lipid metabolism and transport, catabolism, cell growth and death, and environmental adaptation. Scatter plot and volcano plot analysis showed up-regulated and down-regulated genes are shown in Supplementary Figs. S2 and S3.

### Phylogeny tree and conserved motif analysis in selected genes

**Figure 7:**
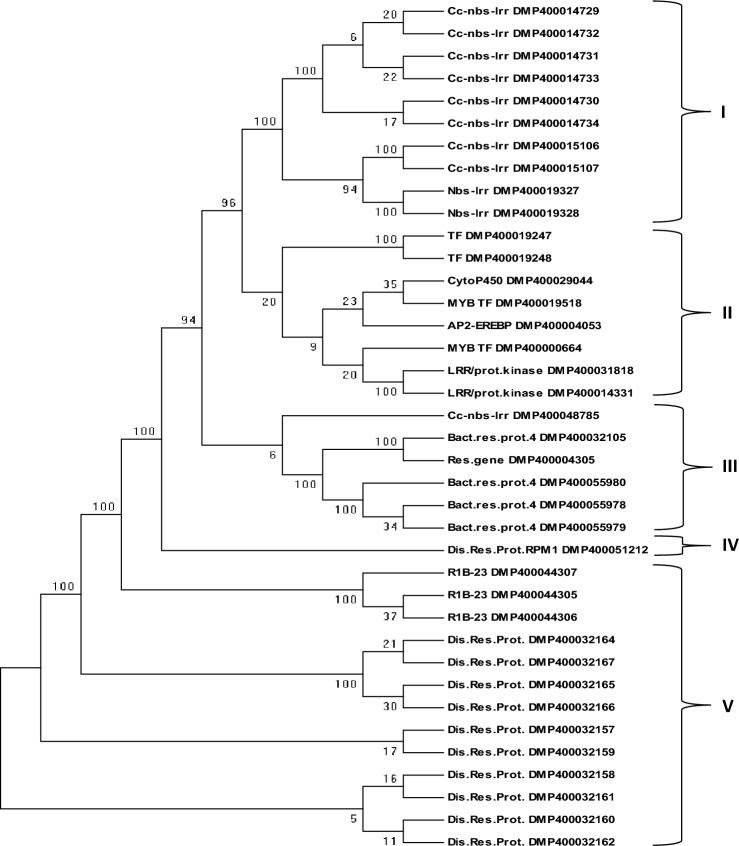
A cluster analysis based on the Neighbor-Joining method derived from the bootstrap consensus tree inferred from 100 replicates using the MEGA software showing the relationship among the 38 amino acid sequence of 18 genes involved in conferring late blight resistance in potato genotypes.

**Figure 8:**
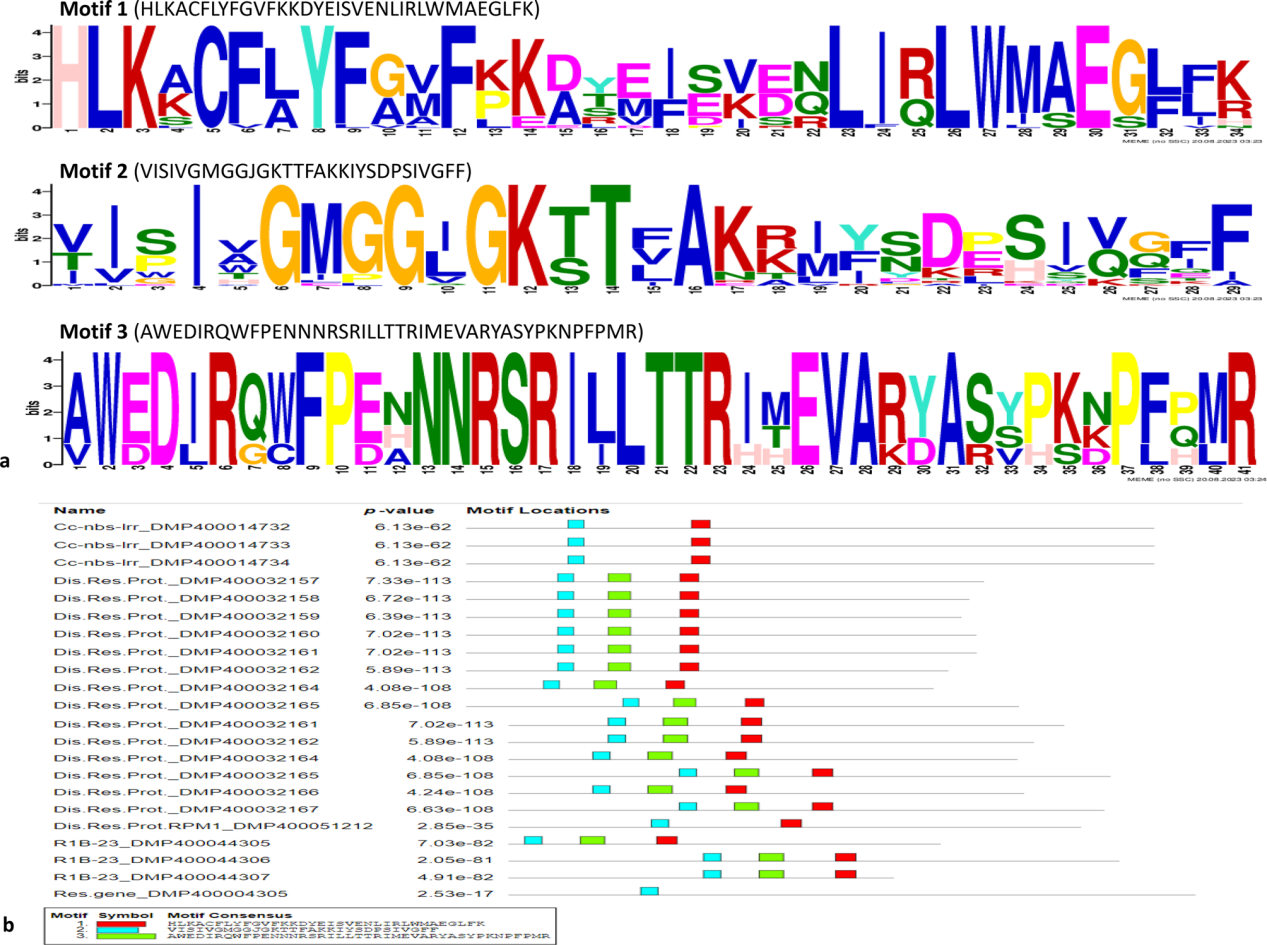
Conserved motifs analysis using the MEME (version 5.5.3) software in 38 amino acid sequences of 18 genes involved in late blight resistance in potato genotypes. (**a**) Sequence logo of three conserved motifs, (**b**) Motif location and gene name (in short) with peptide IDs (prefix with ‘PGSC0003’).

Multiple sequence alignments of 38 amino acid sequences belonging to 18 selected genes were performed (Supplementary Table S5). These selected genes belonged to disease resistance like bacterial spot disease resistance protein 4, disease resistance proteins, disease resistance protein RPM1, late blight resistance protein homolog R1B-23, CC-NBS-LRR resistance proteins, LRR family proteins, NBS-LRR resistance proteins, cytochrome P450, MYB and AP2-EREBP TFs. Subsequently, the aligned amino acid sequences were used for phylogeny analysis using the MEGA software. The Neighbor-Joining tree was constructed, which clearly distinguished the 38 amino acid sequences representing 18 genes into five major clusters (I–V) (Fig. 7). Cluster I contained a total of 10 sequences consisting of CC-NBS-LRR resistance proteins (8) and NBS-LRR resistance proteins (2). Cluster II included 8 seqeunces such as transcription factors (2), cytochrome P450 (1), MYB TF (2), AP2-EREBP TF (1), and LRR family proteins (2). On the other hand, cluster III contained 6 sequences such as CC-NBS-LRR resistance protein (1), resistance gene (1), bacterial spot disease resistance protein 4 (4). Interestingly, cluster IV has only one sequence of disease resistance protein RPM1 (DMG400029405; DMP400051212). Lastly, cluter V possessed total 13 amino acid sequences including late blight resistance protein homolog R1B-23 (3), disease resistance proteins (10). Thus, phylogeny anaysis indicated genetic relationship among the selected resistance genes, TFs and CC-NBS-LRR/NBS-LRR/LRR proteins conferring late blight resistance in potato genotypes. Furthermore, these amino acid sequences were scanned using InterProScan database, and observed common family/domain particularly disease resistance protein, plants (IPR044974) in all resistance genes and CC-NBS-LRR proteins. Whereas, other TF revealed different domains like MYB domain (IPR017930), LRR domain (IPR032675) and AP2/ERF domain (IPR044808). Sequences were also analysed and three conserved motifs alongwith motif locations were predicted using the MEME software (Fig. 8).

### Validation of selected genes by RT-qPCR analysis

Selected eight genes (2 genes from each sample of both up-regulated and down-regulated) were validated by RT-qPCR analysis using Kufri Bahar as highly susceptible control. RT-qPCR analysis was performed for one of each gene from up-regulated and down-regulated DEGs, such as disease resistance protein and C_2_H_2_-type zinc finger protein for P7; gamma aminobutyrate transaminase isoform 2, and AP2/ERF domain-containing transcription factor for Crd6; bacterial spot disease resistance protein 4 and leucine-rich repeat protein in Kufri Girdhari; and disease resistance protein and protein kinase domain-containing protein in Kufri Jyoti. RT-qPCR results are consistent with the RNA-seq results with minor variation in gene expression values (Supplementary Table S6).

## Discussion

### Transcriptome profiling for late blight resistance in potato

We provide an overview of global gene expression profiles for genes associated with late blight resistance in potato by total RNA sequencing. This study included highly resistant interspecific potato somatic hybrids P7 (*S. pinnatisectum*-originated) and Crd6 (*S. cardiophyllum*-originated) and highly resistant potato variety Kufri Girdhari, and popular but susceptible variety Kufri Jyoti compared with highly suscetible Kufri Bahar (control). Genes were analysed after artificial inoculation of *P. infestans* at 96 h post-inoculation stage. Our findings are in consistent with previous results on late blight resistance assays in potato on both cultivated and wild species, including our interspecific somatic hybrids^6,7^. Whole transcriptome analysis has identified genes in contrasting resistant and susceptible genotypes^9,10^. Our RNA-seq results were also in accordance with earlier findings on gene identification through microarray technology in potato cv. Kufri Girdhari^17^. This study provides an overview of molecular signatures of somatic hybrids (P7 and Crd6) and varieties (Kufri Jyoti and Kufri Girdhari) suggesting genetic make-up consisting of a network of resistance genes, TFs and stress related genes. Our study sheds light on improving understanding of the induced genes associated with late blight resistance in potatoes.

### Disease resistance genes play major role in late blight resistance in potato

Disease resistance proteins play an important role in late blight resistance in potato. Resistance (R) proteins in plants mediate the recognition of specific pathogen-derived factors called avirulence (*Avr*) proteins. Upon *Avr* perception, R proteins initiate defence responses that limit further pathogen progress. These responses often result in macroscopically visible cell death, referred to as the hypersensitive response. To illustrate, highly up-regulated genes in resistant genotypes were bacterial spot disease resistance protein 4, CC-NBS-LRR resistance protein, disease resistance protein RPM1, and Sn-2 protein. Earlier, we observed similar group of genes such as disease resistance genes through microarray technology in potato conferring late blight resistance^17,18^.

Plant defense response consists of a multitude of reactions after pathogen infection. The majority of R proteins are NBS-LRR proteins containing a central nucleotide-binding and hydrolysing domain (NB-ARC) and a C-terminal leucine-rich repeat (LRR) domain. Many R genes like putative disease resistance protein over- or under-expressed during host pathogen interaction. It is known that defence-related R gene as well as signal molecules are induced at 72 h post-inoculation, which results in metabolic changes in plants^19^. In this study, 96-h post inoculated samples were analysed due to delay in reaction response between resistant and susceptible genotypes, which might be due to different *P. infestns* strains, controlled environmental conditions and genotype response. Recently, Duan and co-workers^12^ identified higher induction of susceptibility genes such as SWEET after *P. infestans* inoculation on potato. Our results provide a valuable resource for understanding the interactions between *P. infestans* and potato at the 96-h post-inoculation stage.

The Indian potato variety Kufri Jyoti was developed in the year 1960s possessing late blight resistance due to the presence of *R* genes derived from the hexaploid wild species *S. demissum*. But due to the evolution of new strains of *P.* infestans, resistance has broken down in this variety and it is susceptible now. Another newly released potato variety Kufri Girdhari was developed in the year 2008, which contains high resistance to late blight. This is might be due to the presence of several resistance genes as observed in this study, such as CC-NBS-LRR type resistance protein (PGSC0003DMG400008596), disease resistance protein RPM1 (PGSC0003DMG400029405), disease resistance protein (PGSC0003DMG400018464), MYB transcription factor MYB139 (PGSC0003DMG402010883) and late blight resistance protein homolog R1B-23 (PGSC0003DMG400025545). Probably these genes provide late blight resistance in Kufri Girdhari against *P. infestans* infection.

### Transcription factors (TFs) regulates gene expression to provide late blight resistance

TFs are one of the key regulators in plant metabolism. TFs constitute an important part of gene networks and signalling pathways in biotic/abiotic stress response. Indeed, the regulation of defense gene expression is largely governed by specific transcription factors. In this study, highly up-regulated TFs genes in resistant genotypes were MYB, MYB139, AP2-EREBP, and C2H2L TFs, thylene-responsive transcription factor 1B and many other genes. Highly down-regulated TFs were C2H2-type zinc finger protein, AP2/ERF, TSRF1, WRKY, R2R3, leucine-rich repeat protein and others. Previous study witnesses that genes with putative functions of transcription-related, such as heat shock protein transcription factor, zinc finger ring-box protein-like and NAC domain-containing protein NAC22, and MYB44 TF are induced after *P. infestans* inoculation^19^. Our findings are accordance with the previous findings showing that NAC domain protein is a family of plant-specific transcription factors involved in plant development and disease resistance^20^.

The WRKY and MYB TFs have been demonstrated to play key roles in plant responses to stresses, particularly *P. infestans* infection^21,22^. The zinc finger proteins play a pivotal role in the regulation of plant defense mechanisms against *P. infestans* infection. Hypersensitivity plays a major role in the induction of disease resistance pathway, and acts as a downstream signalling pathway for enhancing the systemic resistance in crop plants. This suggests the importance of the transcription elements in activating the defense system during host–pathogen interactions. Our results showed that many genes in potato leaves up-regulated after *P. infestans* infection. This finding provides an overview of the underlying mechanisms related to the modulation and regulation of pathways in response to *Phytophthora* interactions. Consistent with this, previous studies manifested the induced expression of the responsive genes in the late blight resistance associated TFs such as WRKY, ERF, MAPK, bHLH Myc-type TF and NBS-LRR family genes^10^. We also observed differential regulation of MYB, MYB139, AP2-EREBP, C2H2L, and CC-LRR-NBS genes. Thus, our study highlights the role of TFs and other DNA binding proteins in defense response to *P. infestans* in potato.

### Stress-resonsive genes, protein kinases and phytohormones control late blight resistance

Stress-responsive genes play an important role in imparting disease resistance in potato. Some of the stress-responsive genes and kinases in resistant genotypes were cytochrome P450 hydroxylase, serine-threonine protein kinase plant-type, and leucine-rich repeat receptor kinase. The role of cytochrome P450 has been deciphered for enhancing plant resistance via jasmonic acid and ethylene signaling pathways in soybean. Duan and co-workers^12^ identified up-regulation of lignin-forming anionic peroxidase genes that may participate in ethylene-induced defense response against *P. infestans*. The protein kinases mainly calcium-dependent protein kinases (CDPKs) and leucine-rich repeat receptor-like protein kinases (LRR-RKs) determines key functions in pathogen recognition in lentil^23^. In the line of earlier research findings^10–12^, our results confirmed the involvement of signal transduction and stress-responsive genes in activating and maintaining a defense response to *P. infestans* in potato.

The activation of signaling pathways is mostly regulated by salicylic acid, jasmonic acid, ethylene, which induce gene expression and defense response genes against *P. infestans* inoculation in plants^24^. We observed phytohormones related genes in resistant genotypes such as gibberellin regulated protein, BRASSINOSTEROID INSENSITIVE 1-associated receptor kinase 1, ethylene-responsive proteinase inhibitor 1, and ethylene-responsive transcription factor. Our findings were suppoted by Yang et al.^9^ indicating gene expression profiling under exogenous ethylene application in late blight resistant potato genotype SD20. Consequently, they identified multiple signaling pathways including ethylene, salicylic acid, jasmonic acid, abscisic acid, auxin, cytokinin and gibberellin involved in SD20. It has been proven that ethylene-induced gene expression profiling provides insights into the ethylene signaling transduction pathway and its potential mechanisms in disease defense systems in potato. Moreover, the role of photosynthesis is well-known in plant growth and development. Researchers have described that most of the genes associated with photosynthesis pathways were down-regulated upon *P. infestans* inoculation leading to hypersensitive response and leaf lesion^25^. Thus, the stress-responsive genes, protein kinases and phytohormones do play crucial roles in conferring late blight resistance in potato.

## Conclusion

Our study provides a landscape of transcriptome profiling in potato of diverse genetic backgrounds including interspecific somatic hybrid and common potato varieties. This illuminated the role of disease resistance, TFs, stress-responsive genes, and phytohormones genes imparting late blight resistance upon *P. infestans* infection under controlle conditions. We showed that key regulators of late blight resistance are disease resistance genes, TFs, stress-responsive genes and protein kinases. Further, genes were validated by RT-qPCR analysis. Interestingly, gene expression markers were developed for four genes viz*.*, fructose-bisphosphate aldolase (PGSC0003DMG400022263), gamma aminobutyrate transaminase isoform2 (PGSC0003DMG400024281), carbonyl reductase (PGSC0003DMG400000021) and glyceraldehyde-3-phosphate dehydrogenase B subunit (PGSC0003DMG400029406), which showed gene expression in resistant genotypes (P7, Crd6 and Kufri Girdhari) that could be utilized for screenign purpose. However, the functional characterization of these candidate genes would be required in future interventions through transgenics or genome editing technologies. Collectively, our study provides information on genes and regulatory elements involved in late blight resistance in potato and paves a path for its management in future.

## Methods

### Plant materials

In this study, two interspecific potato somatic hybrids namely P7 (*S. tuberosum* + *S. pinnatisectum*)^6^, and Crd6 (*S. tuberosum* + *S. cardiophyllum*)^7^; and three common potato varieties namely Kufri Girdhari, Kufri Jyoti, and Kufri Bahar (control) were used. The genotypes like P7, Crd6 and Kufri Girdhari are highly resistant to late blight, whereas Kufri Jyoti is susceptible and Kufri Bahar (control) is highly susceptible to late blight disease. These genotypes were available at our institute in the Division of Crop Improvement, Indian Council of Agricultural Research—Central Potato Research Insitute, Shimla, Himachal Pradesh, India. Disease-free in vitro plants were maintained by sub-culturing leafy nodes on the Murashige and Skoog (MS) medium^26^ at pH 5.8 supplemented with sucrose (20 g/L) and solidified with gelrite (2 g/L) and cultures were grown at 20 °C under a 16-h photoperiod (light intensity 50–60 µmol/m^2^/s) as described by Sarkar et al.^6^.

### Late blight resistance assay

In vitro plants were grown in the earthen pots (20 × 25 cm^2^) with three replications containing a sterile mixture of soil/FYM-based compost (1:1, v/v) under a glass-house during the summer season under Shimla hills (31.10° N, 7.17° E, 2200 m above mean sea level) at the institute following standard cultural practices. All five genotypes were artificially inoculated with the *P. infestans* isolate HP09/40 (A2 mating type: 1.2.3.4.5.6.7.8.9.10.11) under controlled chamber (18 ± 2 °C temperature, and 80–90% relative humidity). The inoculum was prepared on highly susceptible potato variety Kufri Bahar. The zoospore concentration (5 × 10^4^ sporangia/ml) was adjusted using a hemocytometer. Fifty days old plants were artificially inoculated by mist spray of the pathogen. Late blight symptoms (%) based on leaf and stem lesions were recorded on the leaves and stems after 3, 5 and 7 days of infection. The AUDPC (%.day) was calculated based on the percent disease infestation^27^. Resistant/susceptible genotypes were classified based on the AUDPC value as highly resistant (HR ≤ 50), resistant (R = 50–100), moderately resistant (MR = 100–150) and susceptible (S ≥ 150]^28^. Leaf tissues were collected at 96 h post-inoculation when clear symptoms appeared on the susceptible plants. At this stage, resistant and susceptible plants were clearly distinguished by late blight symptoms. Leaf samples were collected from all five genotypes, snap-frozen in liquid nitrogen, and stored at − 80 °C until further use. The leaf tissues (three biological replicates) were processed for transcriptome sequencing (two technical replicates).

### Transcriptome sequencing and reference mapping

Transcriptome analysis was carried out following our earlier protocols^29^. Briefly, total RNA was isolated from the leaf tissues of five samples using a modified CTAB and lithium chloride method^30^. The isolated RNA was checked for quality on 1% denaturing RNA agarose gel, and quantified by spectrophotometrically using NanoDrop (ThermoFisher Scientific, Wilmington, Delaware USA). The paired-end sequencing libraries were prepared using Illumina TruSeq Stranded mRNA sample prep kit following the manufacturer’s instructions (Illumina, San Diego, CA, USA). The PCR enriched libraries were analyzed on 4200 Tape Station system using high sensitivity D1000 Screen tape as per the manufacturer’s instructions (Agilent Technologies, Santa Clara, CA, USA). The PE illumina libraries were sequenced using Illumina NextSeq500 platform. The raw data was processed using Trimmomatic v0.38 to obtain high-quality reads (QV > 25). The high quality reads were mapped to the reference potato genome^8^ using TopHat v2.1.1 software with default parameters^31^.

### Differential gene expression analysis

The transcriptome data were assembled and DEGs were identified using the cufflinks (v2.2.1) and cuffdiff (version 2.2.1) softwares^32^. DEGs were analyzed in genotypes such as P7, Crd6, Kufri Jyoti, and Kufri Girdhari versus Kufri Bahar (control). Kufri Bahar was used as a control in all DEGs combinations. Log_2_ fold change (FC) values greater than zero were considered up-regulated (≥ 2.00) whereas less than zero were down-regulated (≤ − 2.00) along with a *p* value threshold of 0.05 for statistically significant results. An average linkage hierarchical cluster analysis was performed with the top 50 DEGs using the Multiple experiments Viewer (MeV v4.9.0)^33^. Common genes were identified in DEGs using the Venny 2.1 tool^34^, which were further used for common gene expression marker development based on RT-qPCR analysis in resistant genotypes (Supplementary Table S1). Eurofins Genomics proprietary R scripts were used to depict scatter plots and volcano plots following detailed procedures described elsewhere^29^.

### Gene annotation analysis

The GO annotations of the DEGs were obtained from the Ensembl Plants database for *Solanum tuberosum*. The information on gene counts was assigned to three main GO domains (biological process, cellular component, and molecular function). The bar plots depicting the GO distribution were prepared through the WEGO portal (http://wego.genomics.org.cn/cgi-bin/wego/index.pl)^35^. The functional annotations of the DEGs were carried out against the curated KEGG GENES database using KAAS (KEGG Automatic Annotation Server (http://www.genome.jp/kegg/ko.html)^36^.

### Sequence diveristy and conserved motif analysis in selected genes

A total of 38 amino acid sequences of 18 selected potential genes involved in late blight resistance in potato genotypes were downloaded from the potato genome sequencing consortium^8^ and analysed for sequence diversity and conserved motifs search analysis (Supplementary Table S5). Gene sequences were downloaded from the potato genome sequencing consortium. The sequences were aligned by multiple sequence alignment using BioEdit version 7.2.5 with default parameters^37^. Further, a phylogeny tree was constructed using the Molecular Evolutionary Genetics Analysis 6 (MEGA6) software^38^ based on the Neighbor-Joining method derived from the bootstrap consensus tree inferred from 100 replicates showing the relationship in the selected genes. All the peptide sequences were scanned using the protein function analysis tool InterProScan of EMBL-EBI (https://www.ebi.ac.uk/interpro/)^39^. The Multiple Expectation Maximization for Motif Elicitation (MEME version 4.9.1)^40^ was used to detect conserved motifs in the selected genes.

### Validation of selected genes through RT-qPCR analysis

Eight selected DEGs were validated through RT-qPCR analysis following our earlier protocols^29^. The RT-qPCR primers were designed from the coding sequences of the potato genome with the IDT PrimerQuest Tool (https://eu.idtdna.com/Primerquest/Home/Index) (Supplementary Table S6). The same leaf tissues, as used in transcriptome analysis, were used for RT-qPCR analysis. RT-qPCR analysis was executed using Power SYBR Green PCR Master Mix in ABI PRISM HT7900 (Applied Biosystems Warrington, UK) following temperature profile at 50 °C for 2 min; 95 °C for 10 min; and 40 cycles of 95 °C for 15 s, 60 °C for 1 min, and 72 °C for 30 s with an internal standard potato ubiquitin-ribosomal protein gene (*ubi3*; L22576).

### Accordance statement

This manuscript comply with the ‘IUCN Policy Statement on Research Involving Species at Risk of Extinction’ and the ‘Convention on the Trade in Endangered Species of Wild Fauna and Flora’ for Experimental research and field studies on plants (either cultivated or wild), including the collection of plant material, must comply with relevant institutional, national, and international guidelines and legislation.

### Supplementary Information

Dataset S1.

Dataset S2.

Dataset S3.

Dataset S4.

Dataset S5.

Dataset S6.

Dataset S7.

Dataset S8.

Dataset S9.

Dataset S10.

Dataset S11.

Dataset S12.

Supplementary Information.

## Data Availability

Transcriptome sequence data has been deposited with the NCBI (Bioproject ID: PRJNA836253 and PRJNA744887).

## References

[1] Bradshaw JE, Bryan GJ, Ramsay G (2006). Genetic resources (including wild and cultivated *Solanum* species) and progress in their utilisation in potato breeding. Potato Res..

[2] Hawkes JG (1990). The Potato: Evolution, Biodiversity and Genetic Resources.

[3] Tiwari JK, Devi S, Sharma SA, Chandel P, Rawat S, Singh BP (2015). Allele mining in *Solanum* germplasm: Cloning and characterization of RB-homologous gene fragments from late blight resistant wild potato species. Plant Mol. Biol. Rep..

[4] Jansky S (2006). Overcoming hybridization barriers in potato. Plant Breed..

[5] Tiwari JK, Devi S, Ali N, Luthra SK, Kumar V, Bhardwaj V, Singh RK, Rawat S, Chakrabarti SK (2018). Progress in somatic hybridization research in potato during the past 40 years. Plant Cell Tissue Organ Cult..

[6] Sarkar D, Tiwari JK, Sharma S, Poonam, Sharma SA, Gopal J, Singh BP, Luthra SK, Pandey SK, Pattanayak D (2011). Production and characterization of somatic hybrids between *Solanum*
*tuberosum* L. and *S.*
*pinnatisectum* Dun. Plant Cell Tissue Organ Cult..

[7] Chandel P, Tiwari JK, Ali N, Devi S, Sharma SH, Sharma SA, Luthra SK, Singh BP (2015). Interspecific potato somatic hybrids between *Solanum*
*tuberosum* and *S.*
*cardiophyllum*, potential sources of late blight resistance breeding. Plant Cell Tissue Organ Cult..

[8] Potato Genome Sequencing Consortium (2011). Genome sequence and analysis of the tuber crop potato. Nature.

[9] Yang X, Chen L, Yang Y, Guo X, Chen G, Xiong X, Dong D, Li G (2020). Transcriptome analysis reveals that exogenous ethylene activates immune and defense responses in a high late blight resistant potato genotype. Sci. Rep..

[10] Yang X, Guo X, Yang Y, Ye P, Xiong X, Liu J, Dong D, Li G (2018). Gene profiling in late blight resistance in potato genotype SD20. Int. J. Mol. Sci..

[11] Frades I, Abreha KB, Proux-Wéra E, Lankinen Å, Andreasson E, Alexandersson E (2015). A novel workflow correlating RNA-seq data to *Phythophthora infestans* resistance levels in wild *Solanum* species and potato clones. Front. Plant Sci..

[12] Duan Y, Duan S, Armstrong MR, Xu J, Zheng J, Hu J, Chen X, Hein I, Li G, Jin L (2020). Comparative transcriptome profiling reveals compatible and incompatible patterns of potato toward *Phytophthora infestans*. G3 (Bethesda).

[13] Cao W, Gan L, Shang K, Wang C, Song Y, Liu H, Zhou S, Zhu C (2020). Global transcriptome analyses reveal the molecular signatures in the early response of potato (*Solanum*
*tuberosum* L.) to *Phytophthora*
*infestans*, *Ralstonia*
*solanacearum*, and Potato virus Y infection. Planta.

[14] Gu B, Cao X, Zhou X, Chen Z, Wang Q, Liu W, Chen Q, Zhao H (2020). The histological, effectoromic, and transcriptomic analyses of *Solanum pinnatisectum* reveal an upregulation of multiple NBS-LRR genes suppressing *Phytophthora infestans* infection. Int. J. Mol. Sci..

[15] Gao L, Bradeen JM (2016). Contrasting potato foliage and tuber defense mechanisms against the late blight pathogen *Phytophthora infestans*. PLoS ONE.

[16] Li Y, Zhao D (2021). Transcriptome analysis of scions grafted to potato rootstock for improving late blight resistance. BMC Plant Biol..

[17] Sundaresha S, Tiwari JK, Sindhu R, Sharma S, Bhardwaj V, Chakrabarti SK, Singh BP (2014). *Phytophthora infestans* associated global gene expression profile in a late blight resistant Indian potato cv. Kufri Girdhari. Aust. J. Crop Sci..

[18] Singh R, Tiwari JK, Rawat S, Sharma V, Singh BP (2016). Monitoring gene expression pattern in somatic hybrid of *Solanum*
*tuberosum* and *S.*
*pinnatisectum* for late blight resistance using microarray analysis. Plant Omics.

[19] Sarowar S, Zhao Y, Soria-Guerra RE, Ali S, Zheng D, Wang D, Korban SS (2011). Expression profiles of differentially regulated genes during the early stages of apple flower infection with *Erwinia amylovora*. J. Exp. Bot..

[20] Kikuchi K, Ueguchi-Tanaka M, Yoshida KT, Nagato Y, Matsusoka M, Hirano HY (2000). Molecular analysis of the NAC gene family in rice. Mol. Gen. Genet..

[21] Jiang J, Ma S, Ye N, Jiang M, Cao J, Zhang J (2017). WRKY transcription factors in plant responses to stresses. J. Integr. Plant Biol..

[22] Xiang Q, Judelson HS (2014). Myb transcription factors and light regulate sporulation in the oomycete *Phytophthora infestans*. PloS ONE.

[23] Khorramdelazad M, Bar I, Whatmore P, Smetham G, Bhaaskaria V, Yang Y, Bai SH, Mantri N, Zhou Y, Ford R (2018). Transcriptome profiling of lentil (*Lens culinaris*) through the first 24 hours of *Ascochyta*
*lentis* infection reveals key defence response genes. BMC Genom..

[24] Li YB, Han LB, Wang HY, Zhang J, Sun ST, Feng DQ, Yang CL, Sun YD, Zhong NQ, Xia GX (2016). The thioredoxin *GbNRX1* plays a crucial role in homeostasis of apoplastic reactive oxygen species in response to *Verticillium dahliae* infection in cotton. Plant Physiol..

[25] Burra DD, Lenman M, Levander F, Resjö S, Andreasson E (2018). Comparative membrane-associated proteomics of three different immune reactions in potato. Int. J. Mol. Sci..

[26] Murashige T, Skoog F (1962). A revised medium for rapid growth and bioassays with tobacco tissue culture. Physiol. Plant..

[27] Shaner G, Finney RE (1977). The effect of nitrogen fertilization on the expression of slow-mildewing resistance in Knox wheat. Phytopathology.

[28] Singh BP, Bhattacharyya SK (1995). Field resistance to late blight of four Indian potato cultivars. Potato Res..

[29] Tiwari JK, Buckseth T, Zinta R, Saraswati A, Singh RK, Rawat S, Dua VK, Chakrabarti SK (2020). Transcriptome analysis of potato shoots, roots and stolons under nitrogen stress. Sci. Rep..

[30] Rubio-Pifia JA, Zapata-Peter O (2011). Isolation of total RNA from tissues rich in polyphenols and polysaccharides of mangrove plants. Electron. J. Biotechnol..

[31] Trapnell C, Pachter L, Salzberg SL (2009). TopHat: Discovering splice junctions with RNA-Seq. Bioinformatics.

[32] Trapnell C, Hendrickson DG, Sauvageau M, Goff L, Rinn JL, Pachter L (2013). Differential analysis of gene regulation at transcript resolution with RNA-seq. Nat. Biotechnol..

[33] Howe EA, Sinha R, Schlauch D, Quackenbush J (2011). RNA-Seq analysis in MeV. Bioinformatics.

[34] Oliveros, J. C. V. *An Interactive Tool for Comparing Lists with Venn's Diagrams*. https://bioinfogp.cnb.csic.es/tools/venny/index.html (2007–2015).

[35] Ye J, Fang L, Zheng H, Zhang Y, Chen J, Zhang Z, Wang J, Li S, Li R, Bolund L, Wang J (2006). WEGO: A web tool for plotting GO annotations. Nucleic Acids Res..

[36] Moriya Y, Itoh M, Okuda S, Yoshizawa AC, Kanehisa M (2007). (2007) KAAS: An automatic genome annotation and pathway reconstruction server. Nucleic Acids Res..

[37] Hall TA (1999). BioEdit: A user-friendly biological sequence alignment editor and analysis program for windows 95/98/NT. Nucleic Acids Symp. Ser..

[38] Kumar S, Stecher G, Tamura K (2016). MEGA7: Molecular evolutionary genetics analysis version 7.0 for bigger datasets. Mol. Biol. Evol..

[39] Paysan-Lafosse T, Blum M, Chuguransky S, Grego T, Pinto BL, Salazar GA, Bileschi ML, Bork P, Bridge A, Colwell L, Gough J, Haft DH, Letunić I, Marchler-Bauer A, Mi H, Natale DA, Orengo CA, Pandurangan AP, Rivoire C, Sigrist CJA, Sillitoe I, Thanki N, Thomas PD, Tosatto SCE, Wu CH, Bateman A (2023). InterPro in 2022. Nucleic Acids Res..

[40] Bailey TL, Boden M, Buske FA, Frith M, Grant CE, Clementi L, Ren J, Li WW, Noble WS (2009). MEME SUITE: Tools for motif discovery and searching. Nucleic Acids Res..

