# Supplementary material for: Transcriptome analysis reveals genes associated with late blight resistance in potato
Source: Sci Rep. 2024 Jul 5;14:15501. doi: 10.1038/s41598-024-60608-3 (PMC11226683; doi:10.1038/s41598-024-60608-3)
Supplement: Supplementary file 13 — Supplementary Information. [file 41598_2024_60608_MOESM13_ESM.docx]

**Supplementary Table S1.** Gene expression marker (RT-qPCR) development for selected genes in resistant genotypes

| Sr. No | Gene ID | Gene description | Primer sequence (5’→3’) | Resistant genotype | Gene expression  (Log_2_ FC) | |
| --- | --- | --- | --- | --- | --- | --- |
|  |  |  |  |  | RNA-seq | RT-qPCR |
| 1. | PGSC0003DMG400022263 | Fructose-bisphosphate aldolase | F: GCCATGGACACTGTCATTTTC  R: CTGAATTAGCCTTGCACCTTG | P7 | 3.16 | 2.57 |
|  |  |  |  | Crd6 | 5.16 | 4.82 |
|  |  |  |  | KG | 2.44 | 2.10 |
| 2. | PGSC0003DMG400024281 | Gamma aminobutyrate transaminase isoform2 | F: GAAAGCATTTATCGACAGTCCC  R: GTACCAACACCCCATTCTAGTG | P7 | 2.61 | 2.30 |
|  |  |  |  | Crd6 | 6.69 | 5.98 |
|  |  |  |  | KG | 3.04 | 2.68 |
| 3. | PGSC0003DMG400000021 | Carbonyl reductase | F: AGATCAATTGTGTCTGCCCTG  R: AGAACAAACCAGAAGGACCG | P7 | 2.71 | 2.35 |
|  |  |  |  | Crd6 | 3.21 | 3.10 |
|  |  |  |  | KG | 5.22 | 4.92 |
| 4. | PGSC0003DMG400029406 | Glyceraldehyde-3-phosphate dehydrogenase B subunit | F: TGAGCCTCTTGTGTCAGTTG  R: ATCCCCATTCGTTGTCATACC | P7 | 2.05 | 1.79 |
|  |  |  |  | Crd6 | 4.06 | 3.87 |
|  |  |  |  | KG | 2.96 | 2.48 |

Kufri Bahar was used as control in RT-qPCR study

**Supplementary Table S2: Gene Ontology statistics description**

| Combination | DEGs | Gene Counts | | |
| --- | --- | --- | --- | --- |
|  |  | Biological Process | Cellular Component | Molecular Function |
| P7 | Downregulated | 169 | 113 | 196 |
|  | Exclusive_Control | 134 | 126 | 177 |
|  | Exclusive_Treated | 190 | 142 | 238 |
|  | Expressed_Both | 8,986 | 7,998 | 10,924 |
|  | Upregulated | 184 | 133 | 207 |
|  | **Sub-total** | **9663** | **8512** | **11742** |
| Crd6 | Downregulated | 168 | 109 | 188 |
|  | Exclusive_Control | 161 | 148 | 207 |
|  | Exclusive_Treated | 184 | 136 | 212 |
|  | Expressed_Both | 8,799 | 7,837 | 10,626 |
|  | Upregulated | 131 | 102 | 139 |
|  | **Sub-total** | **9443** | **8332** | **11372** |
| Kufri Jyoti | Downregulated | 122 | 99 | 124 |
|  | Exclusive_Control | 74 | 64 | 83 |
|  | Exclusive_Treated | 124 | 99 | 133 |
|  | Expressed_Both | 8,897 | 7,945 | 10,802 |
|  | Upregulated | 233 | 157 | 274 |
|  | **Sub-total** | **9450** | **8364** | **11416** |
| Kufri Girdhari | Downregulated | 234 | 154 | 258 |
|  | Exclusive_Control | 105 | 85 | 128 |
|  | Exclusive_Treated | 182 | 142 | 194 |
|  | Expressed_Both | 9,155 | 8,147 | 11,189 |
|  | Upregulated | 138 | 110 | 160 |
|  | **Sub-total** | **9814** | **8638** | **11929** |
|  | **Total** | **54476** | **48301** | **65645** |

Kufri Bahar was used as control in gene expression study.

**Supplementary Table S3.** KEGG annotation statistics summary

| Sr. No. | Genotype* | Identified Gene Counts | KEGG Annotated Gene Counts |
| --- | --- | --- | --- |
| 1 | P7 | 19,930 | 5,448 |
| 2 | Crd6 | 19,363 | 5,428 |
| 3 | Kufri Jyoti | 19,496 | 5,414 |
| 4 | Kufri Girdhari | 20,324 | 5,478 |

*Kufri Bahar was used as control in gene expression study.

**Supplementary Table S4.** KEGG Pathways analysis of potato genotypes

| **Sr. No.** | **Pathways** | **Gene Counts** | | | |
| --- | --- | --- | --- | --- | --- |
|  |  | **P7** | **Crd6** | **Kufri Jyoti** | **Kufri Girdhari** |
| Metabolism | | | | | |
|  | Carbohydrate metabolism | 490 | 486 | 485 | 495 |
|  | Energy metabolism | 314 | 312 | 316 | 315 |
|  | Lipid metabolism | 280 | 281 | 272 | 279 |
|  | Nucleotide metabolism | 97 | 96 | 95 | 96 |
|  | Amino acid metabolism | 324 | 326 | 318 | 325 |
|  | Metabolism of other amino acids | 150 | 153 | 152 | 153 |
|  | Glycan biosynthesis and metabolism | 133 | 129 | 131 | 133 |
|  | Metabolism of cofactors and vitamins | 219 | 220 | 221 | 219 |
|  | Metabolism of terpenoids and polyketides | 160 | 158 | 151 | 158 |
|  | Biosynthesis of other secondary metabolites | 173 | 179 | 166 | 176 |
|  | Xenobiotics biodegradation and metabolism | 83 | 82 | 83 | 83 |
| Genetic Information Processing | | | | | |
|  | Transcription | 215 | 214 | 214 | 214 |
|  | Translation | 491 | 492 | 490 | 492 |
|  | Folding, sorting and degradation | 423 | 420 | 426 | 427 |
|  | Replication and repair | 116 | 111 | 118 | 117 |
| Environmental Information Processing | | | | | |
|  | Membrane transport | 28 | 28 | 29 | 28 |
|  | Signal transduction | 646 | 641 | 644 | 651 |
|  | Signaling molecules and interaction | 2 | 2 | 2 | 2 |
| Cellular Processes | | | | | |
|  | Transport and catabolism | 381 | 381 | 386 | 386 |
|  | Cell growth and death | 276 | 273 | 272 | 274 |
|  | Cellular community - eukaryotes | 61 | 61 | 61 | 63 |
|  | Cellular community - prokaryotes | 47 | 47 | 45 | 49 |
|  | Cell motility | 41 | 43 | 41 | 43 |
| Organismal Systems | | | | | |
|  | Environmental adaptation | 298 | 293 | 296 | 300 |

**Supplementary Table S5.** Selected potential genes used for sequence diversity and motif analyses for providing late blight resistance in potato genotypes

| **Sr. No.** | **Gene ID** | **Peptide ID** | **Gene description** |
| --- | --- | --- | --- |
|  | PGSC0003DMG400033334 | PGSC0003DMP400055978  PGSC0003DMP400055979  PGSC0003DMP400055980 | Bacterial spot disease resistance protein 4 |
|  | PGSC0003DMG400018429 | PGSC0003DMP400032105 | Bacterial spot disease resistance protein 4 |
|  | PGSC0003DMG400008596 | PGSC0003DMP400015106  PGSC0003DMP400015107 | Cc-nbs-lrr resistance protein |
|  | PGSC0003DMG400028081 | PGSC0003DMP400048785 | Cc-nbs-lrr resistance protein |
|  | PGSC0003DMG400008394 | PGSC0003DMP400014729  PGSC0003DMP400014730  PGSC0003DMP400014731  PGSC0003DMP400014732  PGSC0003DMP400014733  PGSC0003DMP400014734 | Cc-nbs-lrr resistance protein |
|  | PGSC0003DMG400016616 | PGSC0003DMP400029044 | Cytochrome P450 |
|  | PGSC0003DMG400018462 | PGSC0003DMP400032157  PGSC0003DMP400032158  PGSC0003DMP400032159  PGSC0003DMP400032160  PGSC0003DMP400032161  PGSC0003DMP400032162 | Disease resistance protein |
|  | PGSC0003DMG400018464 | PGSC0003DMP400032164  PGSC0003DMP400032165  PGSC0003DMP400032166  PGSC0003DMP400032167 | Disease resistance protein |
|  | PGSC0003DMG400029405 | PGSC0003DMP400051212 | Disease resistance protein RPM1 |
|  | PGSC0003DMG400025545 | PGSC0003DMP400044305  PGSC0003DMP400044306  PGSC0003DMP400044307 | Late blight resistance protein homolog R1B-23 |
|  | PGSC0003DMG400018264 | PGSC0003DMP400031818 | Leucine-rich repeat family protein / protein kinase family protein |
|  | PGSC0003DMG400008146 | PGSC0003DMP400014331 | Leucine-rich repeat receptor kinase |
|  | PGSC0003DMG400011048 | PGSC0003DMP400019518 | MYB transcription factor |
|  | PGSC0003DMG400000340 | PGSC0003DMP400000664 | MYB transcription factor |
|  | PGSC0003DMG401010943 | PGSC0003DMP400019327  PGSC0003DMP400019328 | Nbs-lrr resistance protein |
|  | PGSC0003DMG400002426 | PGSC0003DMP400004305 | Resistance gene |
|  | PGSC0003DMG400010894 | PGSC0003DMP400019247  PGSC0003DMP400019248 | Transcription factor |
|  | PGSC0003DMG400002272 | PGSC0003DMP400004053 | Transcription factor AP2-EREBP |

**Supplementary Table S6.** RT-qPCR analysis of selected genes

| Sr. No | Gene ID | Gene description | Primer sequence (5’→3’) | Gene expression  (Log_2_ FC) | |
| --- | --- | --- | --- | --- | --- |
|  |  |  |  | RNA-seq | RT-qPCR |
| *P7* | |  |  |  |  |
| 1. | PGSC0003DMG400018462 | Disease resistance protein | F: CATATGGAGCATAGAAGCCTGG  R: GTAGAACACGCATTGGAAAAGG | 5.62 | 4.04 |
| 2. | PGSC0003DMG400021508 | C2H2-type zinc finger protein | F: GCCACCGTGATTTTGACTTG  R: TTGGAGGTAGAGATAGCCGAG | -7.71 | -5.10 |
| *Crd6* | |  |  |  |  |
| 3. | PGSC0003DMG400024281 | Gamma aminobutyrate transaminase isoform2 | F: GAAAGCATTTATCGACAGTCCC  R: GTACCAACACCCCATTCTAGTG | 6.69 | 5.52 |
| 4. | PGSC0003DMG400006369 | AP2/ERF domain-containing transcription factor | F: GGCTAATCCGATGTATACAGGG  R: AACCCCACTCTCCCTCTC | -4.673 | -3.06 |
| *Kufri Girdhari* | |  |  |  |  |
| 5. | PGSC0003DMG400033334 | Bacterial spot disease resistance protein 4 | F: ATGAAGACGGAGTTAGAGATTAGTATG  R: TTTGTAAGATGAGGTTGGAGGTC | 6.99 | 5.42 |
| 6. | GSC0003DMG400030009 | Leucine-rich repeat protein | F: CAGACCCAAACAATGTGCTTC  R: TTCCCAATTGAGGTACCAAGG | -6.11 | -5.23 |
| *Kufri Jyoyi* | |  |  |  |  |
| 7. | PGSC0003DMG400018464 | Disease resistance protein | F: TCCTTCAAGACGCTATTGGC  R: GTCTGATATCTTCCCAGGCTTC | 5.12 | 4.06 |
| 8. | PGSC0003DMG400020660 | Protein kinase domain containing protein | F: GCTTTCCAACTTGCTCAACAG  R: AACCTCAGCATTGTCTCCG | -4.89 | -4.12 |

Kufri Bahar was used as control in the RT-PCR/gene expression study

**
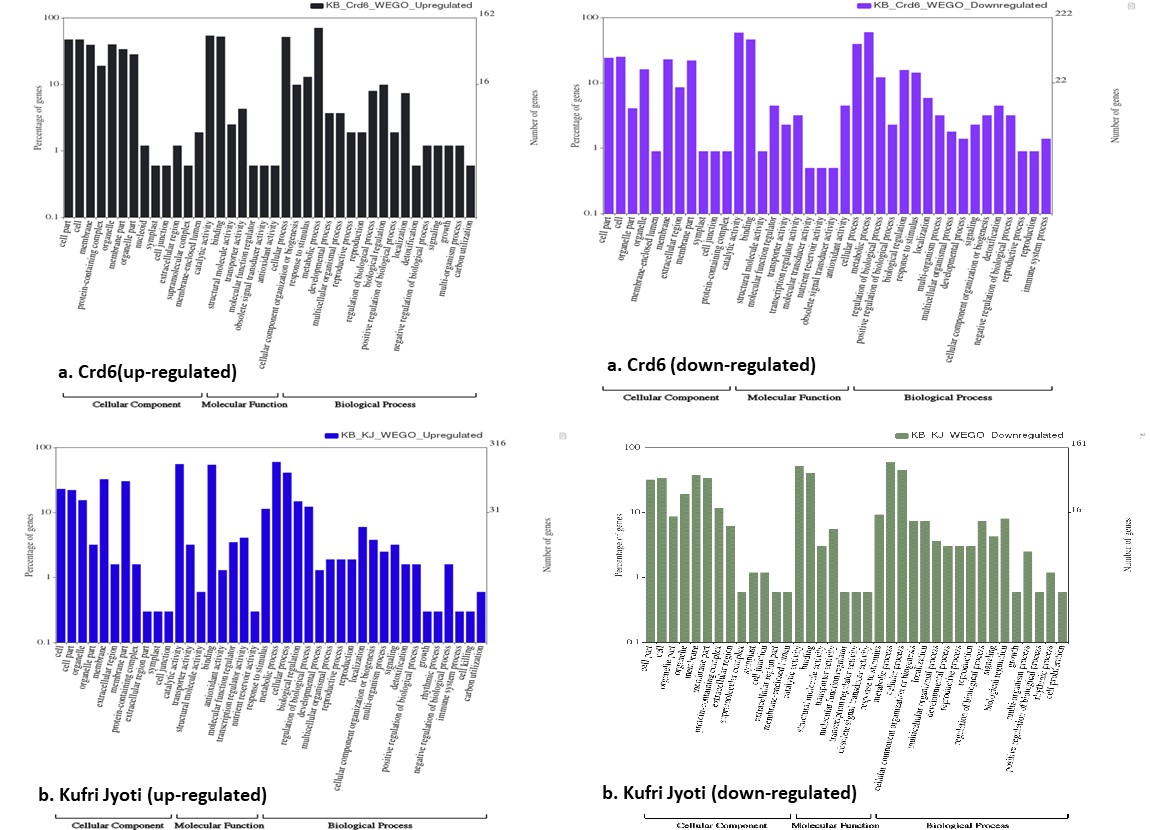
Supplementary Figure S1: GO annotation of genes in Crd6 and Kufri Jyoti**

**Supplementary Figure S2. Scatter plots of genes in P7, Crd6, Kufri Jyoti and Kufri Girdhari**

**
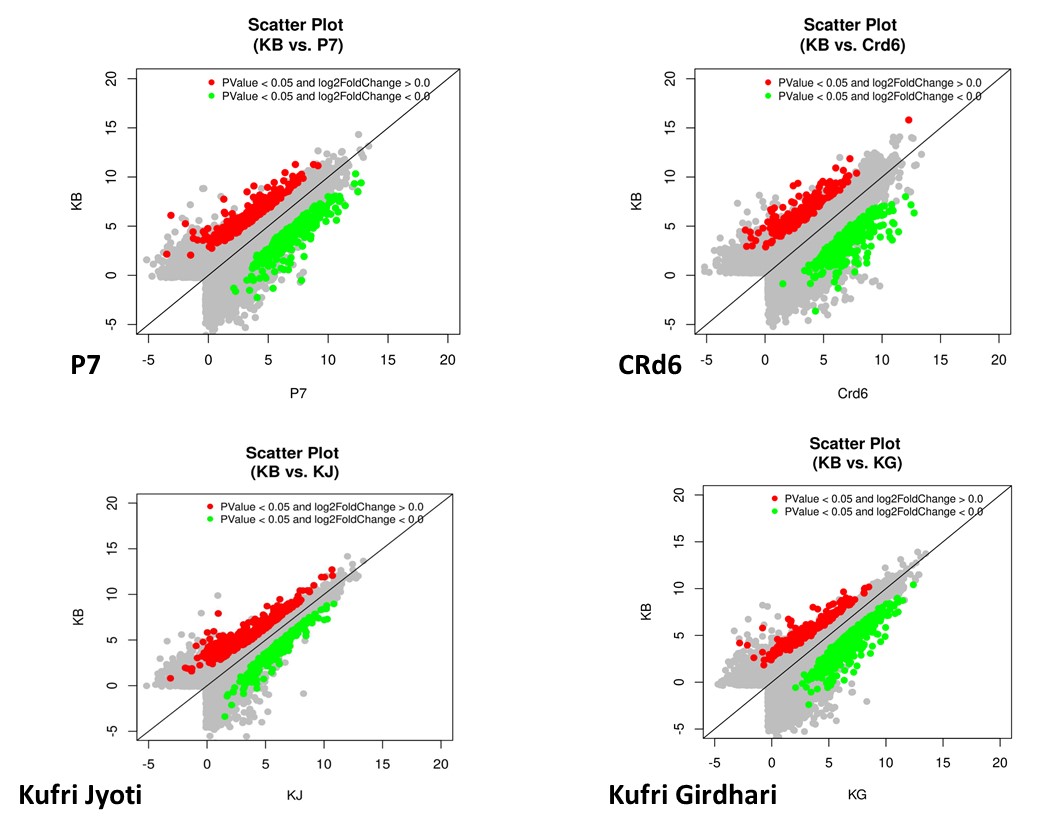
**

**
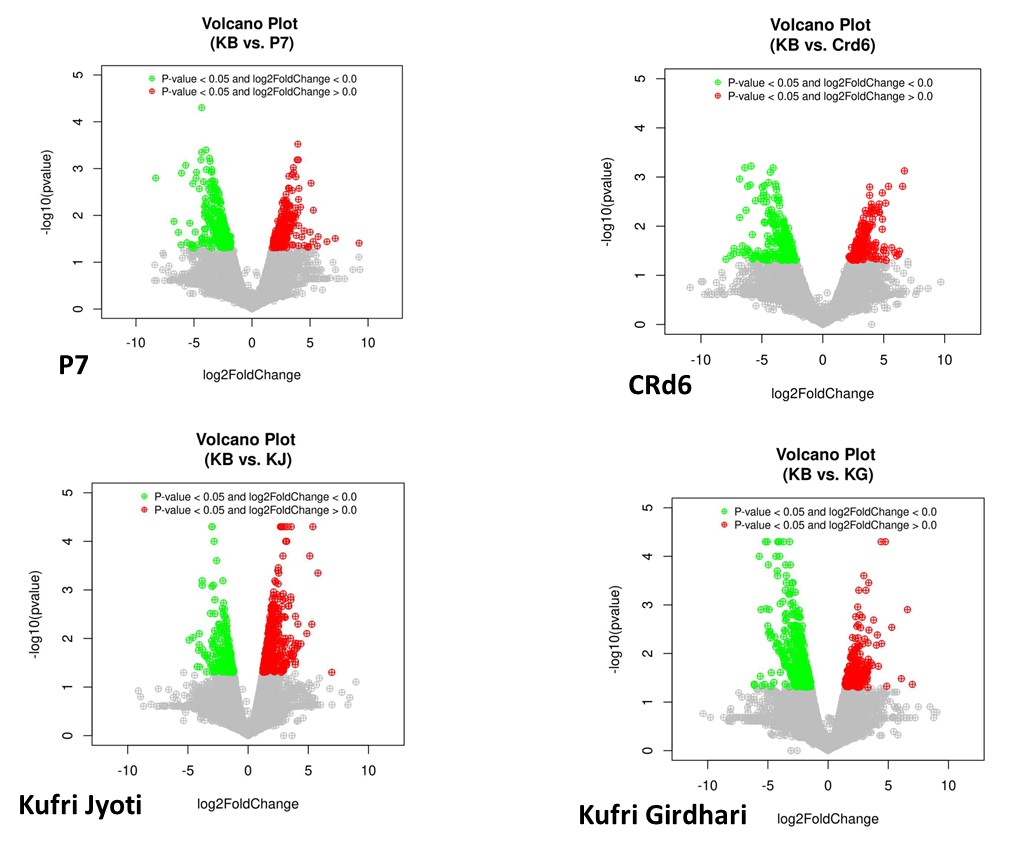
Supplementary Figure S3. Volcano plots of genes in P7, Crd6, Kufri Jyoti and Kufri Girdhari**
